# Determining Bone Turnover Status in Patients With Chronic Liver Disease

**DOI:** 10.7759/cureus.14479

**Published:** 2021-04-13

**Authors:** Tayyaba Bukhari, Lena Jafri, Hafsa Majid, Aysha Habib H Khan, Imran Siddiqui

**Affiliations:** 1 Pathology & Laboratory Medicine, Aga Khan University Hospital, Karachi, PAK

**Keywords:** osteodystrophy, fibrosis, bone resorption, bone remodeling

## Abstract

Introduction

Hepatic osteodystrophy is an osteoporotic bone disease that occurs in chronic liver disease patients. The global prevalence of osteoporosis in patients with chronic liver disease is 30% to 40%. The pathogenesis of hepatic bone disease is not clear, but it occurs due to unstable bone remodeling with increased bone resorption and decreases bone formation. There has been an interest in determining the clinical utility of bone turnover markers (BTMs) in the assessment of osteoporosis in chronic liver patients.

Methods

This was a cross-sectional study conducted in patients with chronic liver disease at the section of chemical pathology, department of pathology and laboratory medicine, Aga Khan University (AKU). A total of 50 patients with age >8 years and a history of liver disease >6 months were recruited from January to October 2019. Liver function tests, i.e. aspartate aminotransferase (AST), alanine transaminase (ALT), albumin, and bilirubin, along with clinical signs of liver disease chronicity, were noted. The samples for BTMs, i.e. total serum alkaline phosphatase (ALP) and serum C-terminal telopeptide of type-1 collagen (CTX) were withdrawn and analyzed on Microlab (ELItech Group, Puteaux, France) and ADVIA Centaur (Siemens Diagnostics, NY), respectively.

Results

The majority of patients were males (n=34, 68%). Twenty-four (48%) patients suffered from fibrosis while 26 (52%) were without fibrosis. Median platelet count (68×10^9^/L (102.5-50)) and median cholesterol levels (102.5 mg/dl (147-99.5)) were decreased, whereas gamma-glutamyl transferase (GGT) levels were higher in the fibrosis group as compared to the non-fibrosis group. The median levels of total ALP were 91.5 IU/L (103-82), and the median levels of CTX were 0.24 pg/ml (0.34-0.21).

Conclusion

In the present study, no significant difference was found in the BTMs of patients with and without chronic liver disease (CLD). However, there was a positive and significant correlation of BTMs, particularly CTX with age, bilirubin levels, and hepatomegaly.

## Introduction

Osteoporosis is the most common bone disorder in patients suffering from chronic liver disease (CLD) and is often known as hepatic osteodystrophy. Osteoporosis is an asymptomatic condition with decreased bone mass, altered bone structure, and low bone strength, which may result in frequent fractures and leads to significant morbidity in this population [1-2]. A literature review on osteoporosis in the Pakistani population by AH Khan et al. showed that the prevalence of osteoporosis range from 5.6% to 17.8% in premenopausal females and 20%-49.3% in postmenopausal females amongst the Pakistani population [3]. CLD is considered a secondary cause of osteoporosis [4-5], as the liver plays a major role in the production of various growth factors and hormones governing many metabolic activities affecting bone. However, the burden of osteoporosis in patients suffering from CLD is not investigated in our population.

Chronic liver disease is one of the most common diseases with a worldwide increase in prevalence and a high risk of irreversible tissue damage leading to cirrhosis. There are multiple etiologies like alcoholism, chronic viral disease, excess tissue iron deposition, immunosuppressive therapy following liver transplantation, etc. [6]. The prevalence of osteoporosis in patients with CLD is 30% to 40% worldwide [7-9]. The pathogenesis of a hepatic bone disease is multifaceted, complex, and difficult to understand but if narrated in simple words, it occurs due to unstable bone remodeling with increased bone resorption and decreased bone formation [10-13].

Liver cirrhosis is associated with high mortality and morbidity. Particularly in developing countries like Pakistan, the rate of progression of viral hepatitis to liver cirrhosis is high, with nearly 41%-52% cases of hepatitis C virus and 30% with hepatitis B virus cases progressing to develop cirrhosis [14]. With therapeutic improvement, the survival of CLD patients has increased, but so has the risk of extra-hepatic manifestations such as osteoporosis. The literature on the status of bone turnover in CLD patients of our population is scarce. The potential socioeconomic burden of developing osteoporosis with increased fracture risk in CLD patients, in a country like Pakistan with a paucity of available health resources, is an enormous challenge and needs increased awareness and early treatment. The current study aimed to determine the association and correlation of bone turnover markers (BTMs) with biochemical markers in CLD patients.

## Materials and methods

It was a cross-sectional study conducted from January to October 2019 in known patients with chronic viral hepatitis, registered at the gastroenterology clinic of Aga Khan University Hospital. Permission from the ethical review committee of Aga Khan University Karachi, Pakistan (ERC # PAT-5105) was taken before the initiation of the study. Only adults (18-65 years of age) with a history of viral hepatitis for more than six months of duration were recruited. While patients with CLD due to any other cause other than viral hepatitis were excluded.

Patients were recruited from the gastroenterology clinic, coming for a fibroscan analysis. Detailed clinical history after informed consent was obtained on a structured questionnaire. Physical examination of patients was performed to note the presence or absence of jaundice, hepatomegaly, ascites, and encephalopathy. Eight to 10 milliliters of the non-fasting blood sample was drawn in serum separator tubes for biochemical analysis of aspartate aminotransferase (AST), alanine transaminase (ALT), gamma-glutamyl transferase (GGT), cholesterol, albumin, total bilirubin, total alkaline phosphatase (ALP) and C-terminal telopeptide of type-1 collagen (CTX). Serum total ALP was analyzed on Microlab 300 (ELItech Group, Puteaux, France) using reagents by Merck & Co. ( Kenilworth, NJ) while serum CTX estimation was done on ADVIA Centaur analyzer (Siemens Diagnostics, NY). All the rest of the biochemical analysis was done on ADVIA 1800 (Siemens Diagnostics). All samples were analyzed in batches with three levels of quality control material run in each batch to validate the results. Patients were also assessed by the Fibroscan analyzer ((Echosens, Paris, France) to determine the extent of fibrosis in the liver. A cutoff of 7.2 was used to identify fibrosis using Fibroscan [15].

Statistical analysis

Data were analyzed in the Statistical Package of the Social Services (SPSS) version 21 (Copyright 1985-2015; IBM Corp., Armonk, NY). The Shapiro-Wilk test was applied to check the hypothesis of normality for quantitative (continuous) variables. Values are supposed to be offered as mean ± standard deviation or median with interquartile range (IQR) for unceasing variables. Qualitative variables were presented by using frequency and percentages. The Mann-Whitney U test was applied to compare the median difference of AST, ALT, albumin, total bilirubin, ALP, and CTX in fibrosis versus non-fibrosis patients. Post-stratification Spearman’s coefficients of correlation test applied to compare the association between both groups. The bone turnover markers were compared with the degree of fibrosis assessed by Fibroscan.

## Results

A total of 50 patients with a history of CLD for more than six months were included in this study, out of which the majority were males (n=34, 68%), and 32% (n=16) were females. Forty-eight percent (n=24) of patients were >40 years of age. The demographics and clinical and biochemical characteristics of patients are further described in Table 1.

**Table 1:** Frequency distribution of clinical and biochemical characteristics of study subjects with chronic hepatitis (n=50) HCV: hepatitis C virus; ALT: alanine transaminase; AST: aspartate aminotransferase; GGT: gamma-glutamyl transferase; ALP: total alkaline phosphatase; CTX: C-terminal telopeptide

Demographics Characteristics	n (%)
Male	34 (68%)
Age <40 years	26(52%)
Fibrosis on fibroscan	24(48%)
Jaundice	2 (4%)
Ascites	3(6%)
Hepatomegaly	1(2%)
Anti HCV positive	35(70%)
HBsAg positive	15(30%)
Elevated serum ALT (Reference range <45 IU/L))	38(76%)
Elevated serum AST (Reference range<35IU/L)	37(74%)
Elevated serum GGT (Reference range <55IU/L)	21(42%)
Elevated serum total bilirubin (0.1-1.2 mg/dl)	6(12%)
Elevated serum direct bilirubin (0.0-0.3 mg/dl)	16(32%)
Elevated serum ALP (Reference range 45-129IU/L)	---
Elevated serum CTX (Reference 16-934 pg/ml)	2 (4%)

There was an almost equal distribution of patients with fibrosis (n=24, 48%) and without fibrosis (n=26, 52%). The majority of the subjects had elevated liver enzymes. However, total ALP and CTX were within the reference range in all subjects. Table 2 shows the comparison between groups with fibrosis and non-fibrosis.

**Table 2:** Comparison of median levels of biochemical markers in liver disease and bone turnover in patients with and without liver fibrosis The Mann-Whitney U-test was applied, at a 95% confidence interval with a 5% level of significance; *p-value is statistically significant. ALT: alanine transaminase; AST: aspartate aminotransferase; GGT: gamma-glutamyl transferase; ALP: total alkaline phosphatase; CTX: C-terminal telopeptide

Study Variables	Overall (n=50)	Patients with fibrosis (n=24)	Patients without fibrosis (n=26)	p-value
Median age in years	40(50-30)	43(52.5-34.5)	38(46-29)	0.085
Median serum bilirubin total(mg/dl)	0.6(0.9-0.4)	0.6(0.95-0.4)	0.6(0.8-0.4)	0.747
Median serum albumin(g/dl)	3.6(4-3)	3.65(4.25-3)	3.55(4-3.2)	0.953
Median serum ALT(IU/L)	48(84-34)	56(89-42)	42.5(54-25)	0.045*
Median serum AST(IU/L)	39.5(49-30)	44(54.5-34)	36(45-25)	0.091
Median serum GGT(U/L)	30(68-15)	50(86-14.5)	26.5(56-15)	0.303
Median serum Cholesterol(mg/dl)	142(169-100)	102.5(147-99.5)	152(188-110)	0.006*
Median platelet count (no./mm3)	118.5(250-70)	68(102.5-50)	237.5(306-130)	<0.001*
Median serum total ALP (IU/L)	91.5(103-82)	95(102-86)	85.5(103-75)	0.248
Median serum CTX (pg/ml)	240(340-210)	230(280-200)	280(490-210)	0.134

The median comparison showed platelet count, serum cholesterol, and serum ALT levels to be statistically significant between the two groups. Median platelet count 68×109/L (102.5-50) and median cholesterol levels 102.5 mg/dl (147-99.5) were decreased, whereas GGT levels were higher in patients with fibrosis. Median levels of total ALP were 91.5 IU/L (103-82) and median levels of CTX were 240 pg/ml (340-210), and the difference was statistically non-significant (Table 2). Table 3 describes the association of biochemical markers with each other.

**Table 3:** Spearman’s coefficients of correlation among CTX, alkaline phosphatase, Fibroscan, and liver function markers Correlation is significant at the 0.01 level (2-tailed)** Correlation is significant at the 0.05 level (2-tailed)* GGT: gamma-glutamyl transferase; ALT: alanine transaminase; AST: aspartate aminotransferase; ALP: total alkaline phosphatase; CTX: C-terminal telopeptide

	Cholesterol	GGT	Platelet Count	Fibroscan	ALT	AST	Total Bilirubin	Albumin	ALP	CTX
Cholesterol	1.000	-.066	.418**	-.501**	-.008	.095	.055	-.158	.193	.161
GGT	-.066	1.000	.109	.173	.227	.139	.176	-.146	.038	-.268
Platelet count	.418**	.109	1.000	-.820**	-.184	-.084	.080	-.154	-.017	.104
Fibroscan	-.501**	.173	-.820**	1.000	.179	.023	-.032	.194	-.045	-.182
ALT	-.008	.227	-.184	.179	1.000	.565**	.303*	-.264	.030	-.368**
AST	.095	.139	.084	.023	.565**	1.000	.437**	-.318*	.020	-.243
Bilirubin total	.055	.176	.080	-.032	.303*	.437**	1.000	-.407**	-.067	-.335*
Albumin	-.158	-.146	-.154	.194	-.264	-.318*	-.407**	1.000	-.267	.086
Alk phosphatase	.193	.038	-.017	-.045	.030	.020	-.067	-.267	1.000	.082
CTX (ng/ml)	.161	-.268	.104	-.182	-.368**	-.243	-.335*	.086	.082	1.000

Liver enzymes, AST and ALT, showed a positive association with each other (r-value 0.56, p-value <0.01). Poor correlation was noted between CTX and other biochemical markers. However, serum CTX showed a weak inverse association that was statistically significant with ALT (r-value -0.36, p-value <0.01) and total bilirubin (r-value -0.33, p-value <0.05).

## Discussion

Literature reports that hepatitis B and C are associated with significantly reduced bone density measurements even in the absence of cirrhotic changes. Viral hepatitis usually presents with increased immune response and release of many resorption-activating cytokines, increasing the duration of liver disease and deteriorating function, further enhancing bone resorption and hence increasing bone loss. Treatment of viral hepatitis with interferon increases the complexity of understanding the cause of bone loss in liver disease [16]. The pathogenesis of hepatic bone disease in patients with CLD is not exactly known; there are multiple factors involved like deficiency of vitamin D and insulin-like growth factor (IGF-1) [17-18], hyperbilirubinemia, and hypogonadism (estrogen and testosterone deficiency), which contribute to bone loss in chronic liver disease.

Findings from the current study show that bone turnover markers (ALP and CTX) were within the reference range in all the studied subjects suffering from chronic hepatitis. Even patients with fibrosis (diagnosed using a Fibroscan cutoff of 7.2) showed values within the reference range. The results of this study also depict that BTMs are not associated with the progression of liver disease or disease severity. CTX and Fibroscan values showed a weak association (r -0.18, p-value >0.05).

Table 4 shows a comparison with other studies in which bone turnover was assessed in liver fibrosis patients. In contrast to our study that shows no increase in bone turnover with normal total ALP and serum CTX levels, bone turnover was found to be increased in all previous studies conducted by Guanabens, Corazza G, George J, and Goral V et al. [19-22]. Normal CTX levels seem to have an association with our population/ethnicity, as in another study conducted by ST Naeem et al., serum CTX levels were found to be normal in postmenopausal Pakistani women with decreased bone mineral density (BMD) on dual-energy X-ray absorptiometry (DXA) scan [23]. The reason for the normal to a very mild increase in CTX in our population needs further investigation and CTX cannot be utilized to predict the development of liver osteodystrophy in the Pakistani population.

**Table 4:** Comparison with other studies regarding bone turnover status in chronic liver disease NAFLD: non-alcoholic fatty liver disease; DKK-1: Dickkopf-related protein-1; RANKL: receptor activator of nuclear factor κβ ligand; PICP: carboxy-terminal propeptide of type I procollagen; PINP: amino-terminal propeptide of type I procollagen; ALP: total alkaline phosphatase; ICTP: telopeptide of type I collagen; TRAP: tartrate-resistant acid phosphatase; HYP: hydroxyproline; PYR: pyridinoline; DPYR: deoxypyridinoline; NTX: type I collagen cross-linked N; CTX: C-telopeptide; UDPD: urinary deoxypyridinoline

Country	Authors	Year	Study subjects	Sample size, n	Bone markers studied and study findings
Italy	A.Mantovani et al	2019	Post-menopausal women with diabetes with NAFLD in 3 groups: No-NAFLD, patients with NAFLD and fibrosis, patients with NAFLD with no fibrosis	77	Bone formation inhibitors: NAFLD and fibrosis showed increased sclerostin levels (54.1 ± 16.4 vs. 36.1 ± 11.9 vs. 42.3 ± 14.7 pmol/L) and decreased serum DKK-1 (26.6 ± 17.8 vs. 49.0 ± 22.4 vs. 2.9 ± 19.4 pmol/L). Resorption markers: NAFLD and fibrosis group had decreased sCTX (0.16 ± 0.09 vs. 0.29 ± 0.17 vs. 0.40 ± 0.28 ng/mL) and increased RANKL (0.04 ± 0.03 vs. 0.08 ± 0.06 vs. 0.11 ± 0.06 pmol/L) compared to other groups.
Turkey	Goral V et al	2010	Liver cirrhosis due to viral hepatitis and primary biliary cirrhosis	85	Formation markers: Increased ALP 109.2 ± 57 IU/L, Decreased osteocalcin 1.05 ± 2.5ng/ml, Resorption markers: Normal UDPD to creatinine ratio 9.4 ± 9.9
India	George J et al.	2009	Viral and alcoholic hepatitis leading to liver cirrhosis	72	Formation markers: Normal ALP 138 ± 61 (132.7) IU&/L, Decreased osteocalcin 2.99 ± 2.5 (1.8) ng/ml, Resorption markers: Increased UDPD to creatinine ratio 11.5 ± 4.5 (12.1)
Italy	Corazza G et al.	2000	Viral hepatitis leading to liver cirrhosis	68	Formation markers: Normal PICP (132 ± 36) ng/ml, increased osteocalcin 11 ± 7 ng/ml, Resorption marker: Increased ICTP (6.6 ± 4.2) ng/ml
Spain	Guanabens et al.	1998	Liver fibrosis due to primary biliary cirrhosis	34	Formation markers: Decreased osteocalcin (16.4 ± 1.2)ng/ml, increased PICP (122.2 ± 5.9) and increased PINP (40.7 ± 5.3) ng/ml, Resorption markers: Increased PYR (65.3 ± 4.4) nM/mM, increased DPYR (9.9 ± 1.1) nM/mM, increased HYP (86.1 ± 6.9) nM/mg, increased NTX (68.9 ± 9.3) nMBCE/mM, and increased CTX (213.5 ± 28.3) ug/mm

There are certain limitations to the study. First, the sample size of the study was small, and another study with a larger sample size in both groups (with and without fibrosis) is further needed. The current study was not longitudinal and bone mineral density using DXA scans was not measured. The serum measurements were taken at a single point thus could miss the associations between inflammatory markers, BTMs, i.e. ALP, CTX, and Fibroscan findings, as the increased inflammatory response to viral infection can contribute to more bone loss. The information about the stage of liver cirrhosis was not available. Additionally, more BTM can be added in future studies to understand the clinical utility of these BTM in chronic hepatitis patients who are at risk of hepatic osteodystrophy.

## Conclusions

In the present study, no significant difference was found in the BTMs of patients with and without CLD. However, there was a positive and significant correlation of BTMs, particularly CTX, with age, bilirubin levels, and hepatomegaly. In conclusion, bone loss in liver disease is still under question, and the role of BTMs for assessing early bone loss in liver disease is uncertain and needs further investigation. The role of systematic inflammation and its association with hepatic osteodystrophy should be considered and investigated.
